# A Text-Book on Gonorrhœa and Its Complications

**Published:** 1914-03

**Authors:** 


**A Text-Book on Gonorrhœa and its Complications.** By Dr. GEORGES LUYS. Translated and Edited by ARTHUR FOERSTER, M.R.C.S., L.R.C.P. (Lond.). Pp. xix, 384. London: Baillière, Tindall & Cox. 1913. Price 15s. net.

It seems to us particularly opportune that this very complete text-book on gonorrhœa has appeared in this country at a time when venereal diseases are receiving special attention both from the medical profession and the general public. Its appearance is also opportune because the study of syphilis and its treatment has of late absorbed so much attention, that that of gonorrhœa and its complications has been overshadowed. Yet from the social point of view, as well as from that of the individual victims, gonorrhœa is a disease of the first importance. Too long have many of the male victims of the disease been prone to regard it lightly, though death is a possible result, and lifelong disability a fairly common one. Too often have they carelessly, ignorantly, or culpably infected the innocent; have given, for example, quoting Luys, their young wife "in exchange for her virginity a poison which may cripple or kill her." "It is pitiful to see the pale faces, the anxious and worn looks, the hollow eyes of these poor young women who suffer permanently from internal pains until they submit to a surgical operation, which gives them the desired relief, but condemns them to complete sterility."

Have not we, the members of the medical profession, been too largely responsible for the ignorance, apathy, and carelessness of the public in this matter? Have the teachers in our medical schools adequately considered gonorrhœa, adequately treated its victims, adequately taught their students what the treatment should be? Have they insisted that our hospitals and infirmaries shall be adequately equipped for such treatment? It is our opinion that with a few notable exceptions they have not. These things are better done on the Continent. We hope that the volume before us will stimulate the younger generation of teachers in our hospitals to greater efforts in these directions.

We believe this work to be the most complete in our language on its subject. The text is lucid and precise, and the illustrations numerous and helpful. Alike to author, translator and publisher we tender our thanks for the production of a most valuable text-book.

